# Matrix protease production, epithelial-to-mesenchymal transition marker expression and invasion of glioblastoma cells in response to osmotic or hydrostatic pressure

**DOI:** 10.1038/s41598-020-59462-w

**Published:** 2020-02-14

**Authors:** Wenjun Pu, Jiawen Qiu, Gregory J. Riggins, Marie-Odile Parat

**Affiliations:** 1 University of Queensland School of Pharmacy, PACE, 20 Cornwall Street, Woolloongabba, QLD 4102 Australia; 2 Department of Neurosurgery, Johns Hopkins University School of Medicine, Baltimore, MD 21213 USA

**Keywords:** CNS cancer, CNS cancer

## Abstract

Both hydrostatic and osmotic pressures are altered in the tumour microenvironment. Glioblastoma (GBM) is a brain tumour with high invasiveness and poor prognosis. We hypothesized that physical and osmotic forces regulate glioblastoma (GBM) invasiveness. The osmotic pressure of GBM cell culture medium was adjusted using sodium chloride or water. Alternatively, cells were subjected to increased hydrostatic force. The proteolytic profile and epithelial–mesenchymal transition (EMT) were investigated using zymography and real-time qPCR. The EMT markers assessed were Snail-1, Snail-2, N-cadherin, Twist and vimentin. Invasion was investigated *in vitro* using extracellular matrix-coated Transwell inserts. In response to osmotic and mechanical pressure, GBM cell lines U87 and U251 and patient-derived neural oncospheres upregulated the expression of urokinase-type plasminogen activator (uPA) and/or matrix metalloproteinases (MMPs) as well as some of the EMT markers tested. The adherent cell lines invaded more when placed in media of increased osmolality. Therefore, GBM respond to osmotic or mechanical pressure by increasing matrix degrading enzyme production, and adopting a phenotype reminiscent of EMT. Better understanding the molecular and cellular mechanisms by which increased pressure promotes GBM invasiveness may help to develop innovative therapeutic approaches.

## Introduction

Physical solid and fluid forces play a key role when solid tumours grow, progress and also respond to therapy^1^. Compressive stresses affect cancer cells by promoting invasiveness and metastasis^2^. Tumours are generally hypoperfused, and interstitial fluid pressure is increased compared to normal tissue^1,3,4^ with both increased hydrostatic pressure^4^ and oncotic pressure^5,6^. Increased interstitial fluid pressure results from abnormal blood and lymphatic vessels, fibrosis and contraction of the matrix by stromal cells^7^. In addition to these stresses common to most solid tumours, brain tumours experience pressure when the tumour grows within a space limited by the skull^8^.

Glioblastoma is the most common primary brain cancer. The average survival time is approximately one year after diagnosis. A major feature of GBM that contributes to its poor prognosis is its high invasiveness. The urokinase-type plasminogen activator (uPA) derives its name from its ability to activate plasminogen into plasmin. While tissue-type plasminogen activator (tPA) plays a role in the fibrinolytic process, uPA is involved in cell migration and tissue remodelling, thereby playing a major role in cancer development and spreading. This role is especially crucial in glioblastoma^9–11^. Equally important and complementary to the uPA system, MMPs play a key role in the control of the tumour microenvironment and ECM, thereby modulating tumor growth, angiogenesis, invasion and metastasis. Recently reports showed that the MMPs play pivotal roles in the invasiveness of GBM by degrading surrounding tissue, activating signal transduction, releasing ECM-bound growth factors, activating growth factors, increasing tumour cell motility, and promoting angiogenesis^12–15^. Multiple studies have reported that the expression of higher level of MMPs in brain tumours is associated with increased tumour aggressiveness^16–18^. Of note, MMP-2 and -9 play a key role in high grade gliomas^19^.

There is limited evidence that GBM cells subjected to compressive strain showed increased mRNA expression of both uPA and uPA receptor. We hypothesized that pressure characteristic of the GBM microenvironment, i.e. dysregulated osmotic and mechanical pressure, promote GBM cell invasiveness.

## Results

### Hyperosmolarity increases GBM matrix protease production

**Figure 1:**
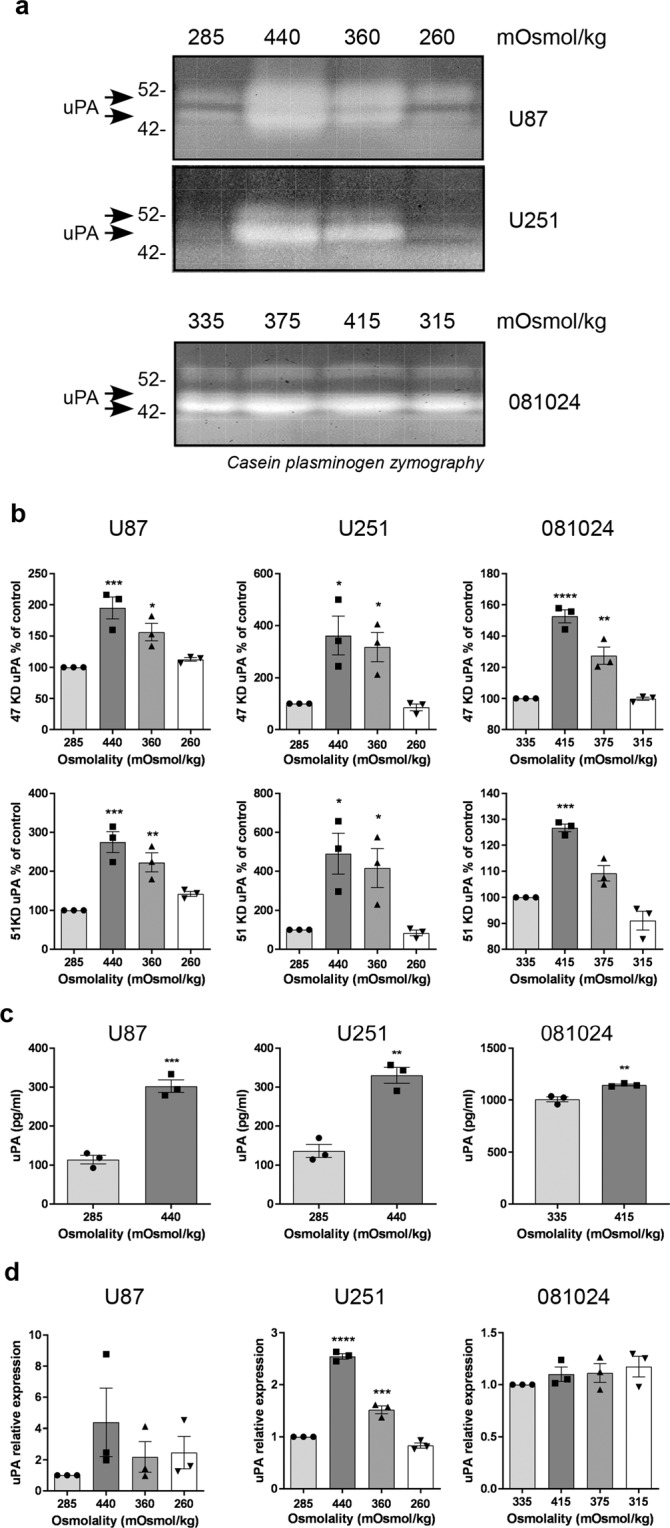
Effect of osmotic pressure on uPA production by GBM adherent and neurosphere cells. (**a**) Conditioned media of cells exposed to control (285 mOsmol/kg) hyper (440 or 360 mOsmol/kg) or hypoosmotic stress (260 mOsmol/kg) were analysed by casein plasminogen zymography. (**b**) Densitometric quantitation of the 47 KD and 51 KD bands corresponding to uPA (**c**) ELISA quantification of uPA in the conditioned medium of GBM cell lines subjected to normo- or hyper-osmolality (**d**) Effect of osmotic pressure on uPA mRNA expression. All results are expressed as mean ± SEM of n = 3 independent experiments, *p < 0.05, **p < 0.01, ***p < 0.001, ****p < 0.0001, one way ANOVA analysis with Dunnett’s multiple comparisons test.

To test the effect of hyper or hypo-osmotic stress on matrix protease production in cell-conditioned medium, cell viability in response to increased (by addition of NaCl) or decreased (by addition of water) osmolality over 6, 12, 24 and 48 h was assessed in preliminary experiments (Supplementary Fig. S1). The osmolalities chosen for further experiments were 285 (control serum-free RPMI), 440, 360 and 260 mOsmol/kg for the U87 and U251 cell lines and 335 (control NeuroCult NS-A Proliferation medium) 415, 375 and 315 mOsmol/kg for the 081024 oncospheres as these did not significantly affect cell viability at 48 h. The urokinase type plasminogen activator (uPA) production was tested using casein plasminogen zymography of the 48 h conditioned medium (Fig. 1a). While hypoosmotic stress had essentially no effect on uPA production, there was an increase in uPA in response to hyperosmolality which was more dramatic in the conditioned medium of the adherent cell lines compared to that of the oncospheres. Densitometric quantification revealed that uPA was increased 2–3 fold in U87, 3–4 fold in U251, and by ~50% in 081024 cells (Fig. 1b) by the highest hyper-osmolality (440 and 415 mOsmol/kg for adherent and oncosphere cells, respectively). The increased uPA production under hyperosmolar conditions was confirmed using ELISA quantification of uPA in the conditioned medium (Fig. 1c). Furthermore, the expression of uPA mRNA was tested in cells after 48 h exposure to hyper or hypoosmotic stress and confirmed increased expression of uPA in the adherent cells (although statistical significance was reached only with the U251 cells) (Fig. 1d). In contrast, the mRNA expression of uPA receptor was unchanged (data not shown). Increased mRNA expression of uPA in 415 mOsmol/kg medium was confirmed in two additional oncosphere cell lines, JHH136 and 2010.016 A (Supplementary Fig. S2).

**Figure 2:**
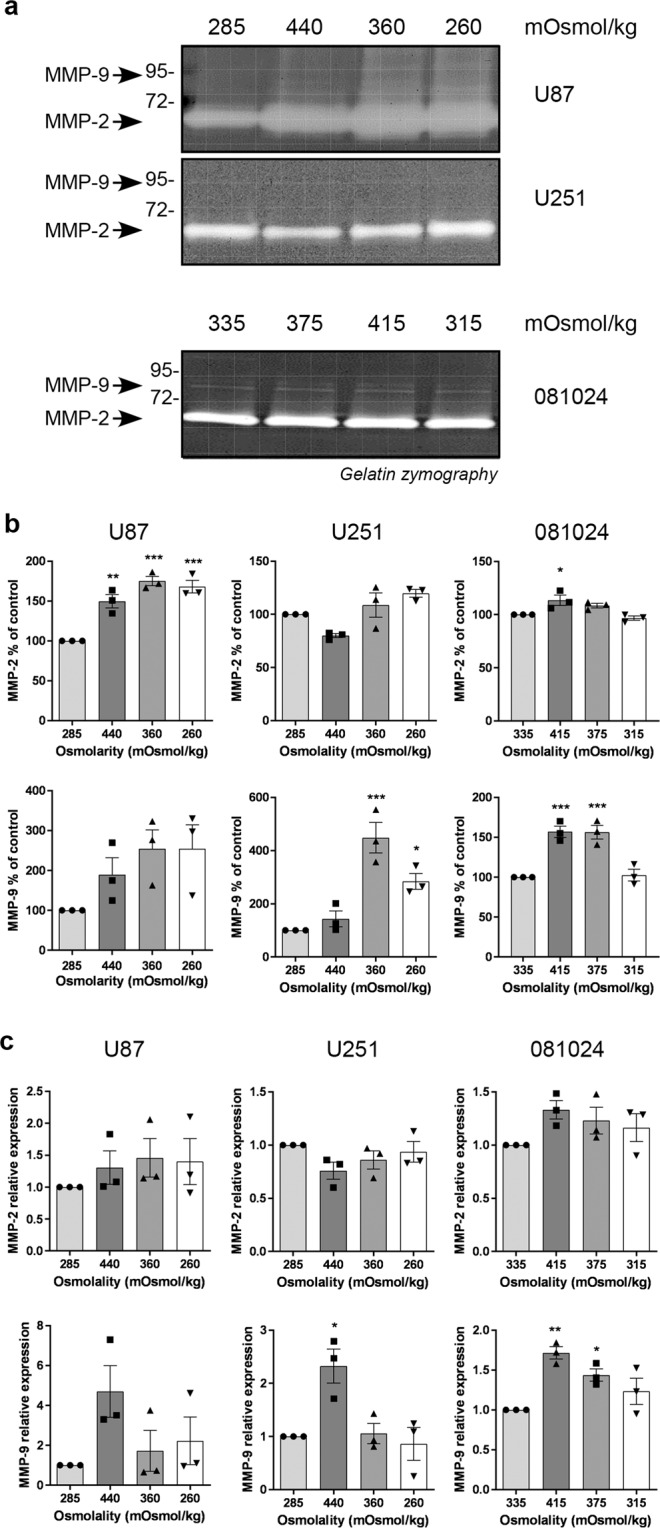
Effect of osmotic pressure on MMP-2 and MMP-9 production by GBM adherent and neurosphere cells. (**a**) Conditioned media of cells exposed to control (285 mOsmol/kg) hyper (440 or 360 mOsmol/kg) or hypoosmotic stress (260 mOsmol/kg) were analysed by gelatin zymography. (**b**) Densitometric quantitation of MMP-2 and MMP-9 produced in the conditioned medium. (**c**) Effect of osmotic pressure on MMP-2 and MMP-9 mRNA expression. All results are expressed as mean ± SEM of n = 3 independent experiments, *p < 0.05, **p < 0.01, ***p < 0.001, one way ANOVA analysis with Dunnett’s multiple comparisons test.

We further assessed the production of gelatinases in GBM cells exposed to osmotic stress. At least one gelatinase was increased in the conditioned medium of all cells exposed to hyperosmotic media (Fig. 2a,b and Supplementary Fig. S3a). In addition, hypoosmotic stress increased MMP-2 in U87 cells and MMP-9 in U251 cells. An increased expression of MMP-9 mRNA was seen in adherent cells at 440 mOsmol/kg and in the 081024 oncospheres at 375 and 415 mOsmol/kg (Fig. 2c). Together these results indicate that GBM cells respond to osmotic pressure by an increase in matrix degrading enzyme expression, and the intensity of this response is cell line- and enzyme-specific.

### Hyperosmolarity-induced matrix protease production is not a common response of all cancer cells

We next tested whether non GBM cancer cells similarly responded to increased osmolality by producing more uPA, MMP-2 and MMP-9. We tested the response of the prostate cancer cell line DU145, and breast cancer cell lines MDA-MB-231 and MDA-MB-468 (Supplementary Fig. S4). While DU145 cells grow in RPMI medium and the addition of 1 and 2% V/V 5 M NaCl results in an increase of osmolality from 285 to 360 and 440 mOsmol/kg, respectively, the MDA-MB-231 and -468 cells grow in DMEM and the addition of 1 and 2% V/V 5 M NaCl results in an increase of osmolality from 350 to 430 and 510 mOsmol/kg, respectively. Analysis of the conditioned medium by casein plasminogen zymography (Supplementary Fig. S4a) and gelatin zymography (Fig. S4 b) did not reveal any increase in proteases. On the contrary there decreased uPA was apparent in breast cancer cells MDA-MB-231 and -468 at 510 mOsmol/kg and decreased MMP-2 production in MDA-MB-468 at both 430 and 510 mOsmol/kg. MMP-9 was undetectable in these cell lines. These results indicate that pressure-induced increase in matrix degrading proteases is not a feature of all cancer cell lines.

### Hyperosmolarity promotes GBM invasiveness

**Figure 3:**
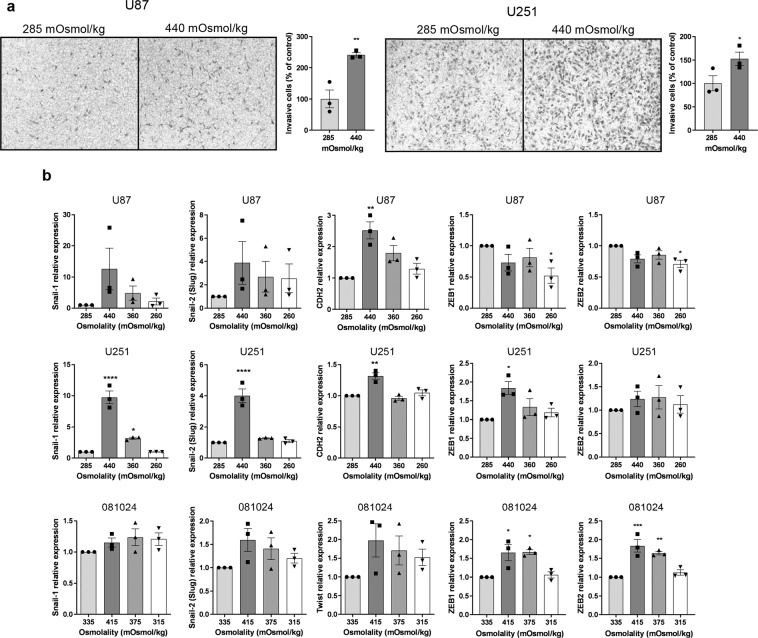
Effect of osmotic pressure on U251 and U87 *in vitro* invasion potential. (**a**) Cell invasion through Matrigel-coated Transwells was determined in control (285 mOSmol/kg) or hyperosmotic (440 mOsmol/kg) media. Representative micrographs with crystal violet-stained cells are shown. Quantitation of invaded cells is shown as percent of normo-osmotic control. Results are expressed as mean ± SEM of n = 3 independent experiments, *p < 0.05, **p < 0.01, unpaired Student t test. (**b**) mRNA expression of EMT markers in U87 U251 and 081024 cells after 48 h of exposure to control (285 mOsmol/kg for adherent cells, 335 mOsmol/kg for oncospheres) hyper or hypoosmotic stress. All results are expressed relative to cells incubated in normo-osmotic medium as mean ± SEM of n = 3 independent experiments, *p < 0.05, **p < 0.01, ****p < 0.0001, one way ANOVA analysis with Dunnett’s multiple comparisons test.

To test whether osmotic stress-induced changes in matrix protease production translated into increased invasiveness, we subjected cells to osmotic stress while invading through extracellular matrix protein-coated inserts. Representative images of stained cells on the lower surface of filters and quantification of the number of invaded cells (Fig. 3a) clearly show that hyperosmotic stress promotes invasion of GBM adherent cell lines. Invasiveness is one of the features of the mesenchymal phenotype that is adopted by aggressive tumor cells including gliomas^20^. We assessed the expression of EMT markers^20–23^ Snail-1, Snail-2 (Slug), Twist, vimentin N-cadherin (CDH2), ZEB1 and ZEB2 in cells exposed to control or hyperosmotic medium for 48 h. The mRNA expression of EMT markers Snail-1, Slug and N-cadherin was increased by exposure of the adherent cell lines to hyperosmolar media (albeit statistical significance was seen for only some of the markers) while ZEB1 and ZEB2 were induced by hyperosmolarity in the 081024 oncospheres (Fig. 3b). Snail-1, Slug and N-cadherin as well as ZEB1 and ZEB2 were increased in the 2010.016 A oncosphere cell line (Supplementary Fig. S2). Therefore our results indicate that GBM cells respond to osmotic pressure by an increase in EMT marker expression, with cell line and marker specificity.

### Hydrostatic pressure increases matrix protease production and EMT marker expression by GBM cells

**Figure 4:**
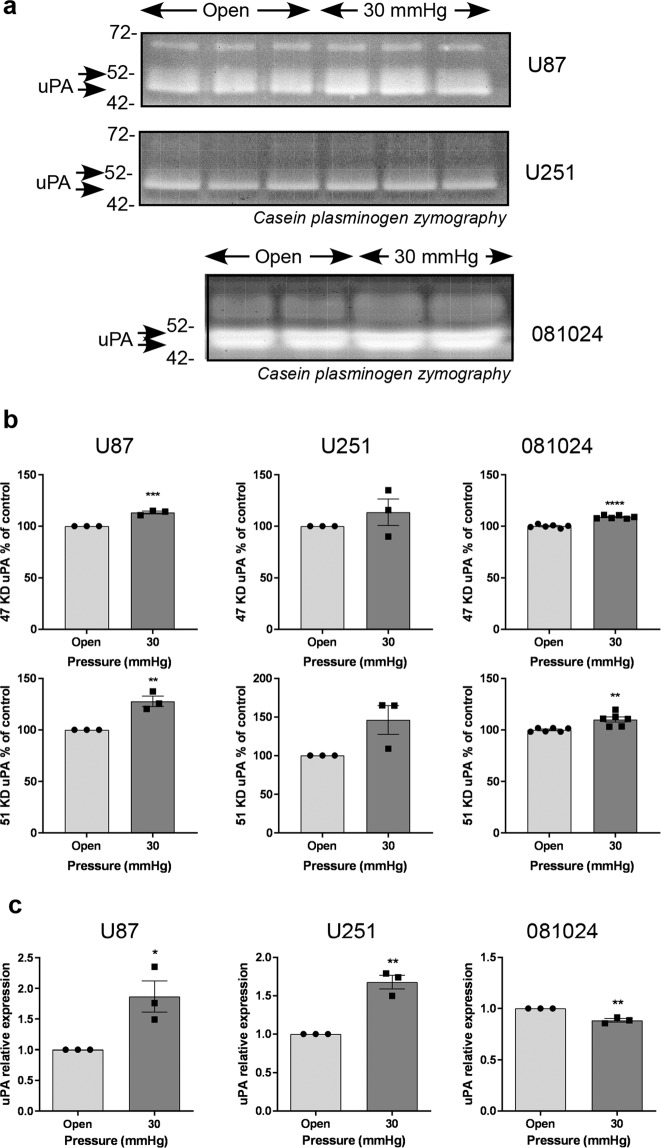
Effect of hydrostatic pressure on uPA production by GBM adherent and neurosphere cells. (**a**) Conditioned media of cells exposed to hydrostatic pressure for 48 h were analysed by casein plasminogen zymography. (**b**) Densitometric quantitation of the 47 KD and 51 KD bands corresponding to uPA. (**c**) Effect of hydrostatic pressure on uPA mRNA expression. All results are expressed as mean ± SEM of n = 3 independent experiments, *p < 0.05, **p < 0.01, ***p < 0.001, ****p < 0.0001, unpaired Student t test.

**Figure 5:**
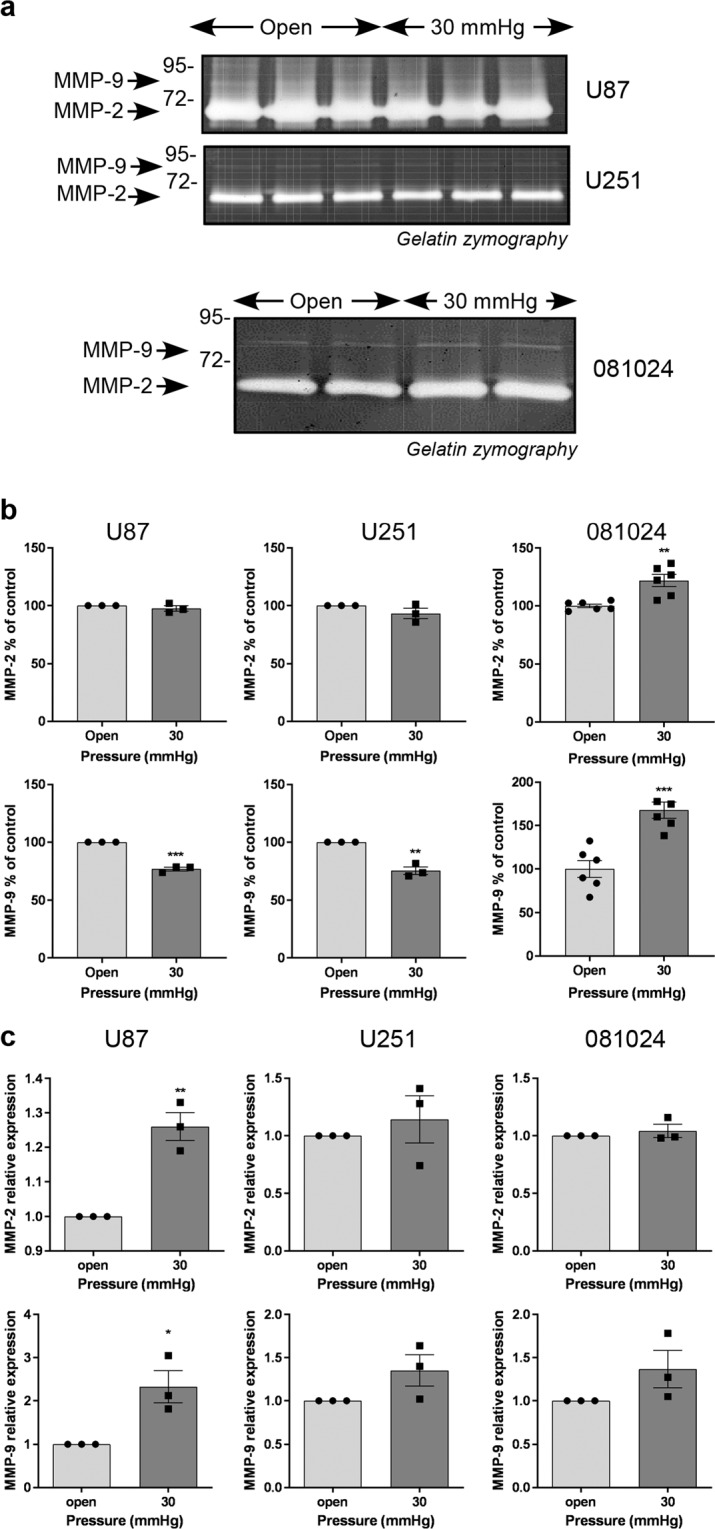
Effect of hydrostatic pressure on matrix metalloproteinase production by GBM adherent and neurosphere cells. (**a**) Conditioned media of cells exposed to hydrostatic pressure for 48 h were analysed by gelatin zymography. (**b**) Densitometric quantitation of MMP-2 and MMP-9 produced in the conditioned medium. (**c**) Effect of hydrostatic pressure on MMP-2 and MMP-9 mRNA expression. All results are expressed as mean ± SEM of n = 3 independent experiments, *p < 0.05, **p < 0.01, ***p < 0.001, unpaired Student t test.

**Figure 6:**
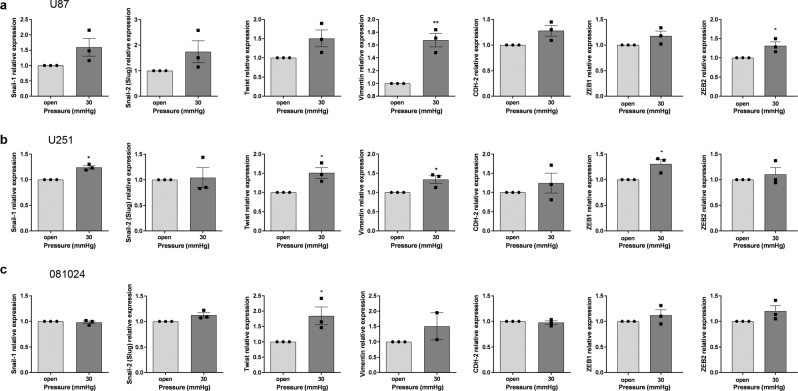
Effect of hydrostatic pressure on EMT marker expression by GBM adherent and neurosphere cells. The mRNA expression of EMT markers was quantified using qRT-PCR in (**a**) U87 and (**b**) U251 cells after 48 h of hydrostatic pressure as indicated. (**c**) The mRNA expression of EMT markers was quantified using qRT-PCR in oncosphere cells after 48 h of hydrostatic pressure as indicated. All results are expressed as mean ± SEM of n = 3 independent experiments, *p < 0.05, **p < 0.01, unpaired Student t test.

GBM cells are subjected to increasing interstitial fluid pressure as the tumour grows^24^. We tested the effect of increased hydrostatic pressure on matrix protease production. Cells exposed to 30 mmHg over 48 h exhibited a slight increase in uPA production in the conditioned medium, which was statistically significant for U87 cells and 081024 oncospheres (Fig. 4a,b). This increase was also seen at the mRNA level in the adherent cell lines (Fig. 4c). Gelatin zymography detected an increased production of MMP-2 and MMP-9 only in the oncospheres (Fig. 5a,b) while increased MMP-2 and MMP-9 mRNA was apparent in U87 cells only (Fig. 5c). The setup employed did not allow us to test the effect of increased hydrostatic pressure on invasion through BME-coated inserts (Transwells), however we quantified the expression of the EMT markers Snail-1, Snail-2 (Slug), Twist, vimentin N-cadherin (CDH-2) ZEB1 and ZEB2 in cells exposed to 30 mmHg compared to control cells (Fig. 6). Expression of these markers was inconsistently increased (although only by ~50%) in adherent (Fig. 6a,b) and oncosphere (Fig. 6c) cells. These results show that hydrostatic pressure increased at clinically relevant levels *in vitro* enhances some EMT markers in GBM cells.

## Discussion

Our experiments show that GBM cells can respond to pressure by increasing their invasive potential. Tumours are exposed to alterations in hydrostatic pressure^4^ and oncotic pressure^5,6^. In addition, as GBM grows within the confines of the skull, GBM cells are exposed to elevated intracranial pressure compressing the tumour. It has long been known that intracranial pressure is increased in patients with brain tumours as measured *via* cerebrospinal fluid pressure (CSFP). In 18 patients with primary or metastatic brain tumours, mean cerebrospinal fluid pressures of ~30 mmHg were recorded, with plateau waves of up to 100 mmHg^8^. In comparison, CSFP at lumbar puncture in patients lying down on their side is considered normal at ~10 mmHg^25^, with normal values proposed to be 4.4 to 18.4 mmHg^26^. The hydrostatic pressure that cells are exposed to in our study (30 mmHg) is thus clinically relevant. The tumour microenvironment is also characterized by high interstitial colloid osmotic pressure^7^. Using magnetic resonance spectroscopy, the concentration of myoinositol, an organic osmolyte indicative of cell metabolic reaction to osmotic changes into the brain, was shown to be lower in GBM compared to control tissue, to increase after bevacizumab treatment (which normalizes the vasculature and reduces the oedema), and to correlate with better overall survival of the patients^27^. Interstitial fluid pressure reduction (via strategies such as induction of endogenous antisecretory factor (AF) or administration of exogenous AF peptide) improved the outcome of GBM in patient-derived xenograpft mouse experimental models^24^ and blocked compression-induced proliferation of GBM oncospheres^24^.

Hyper and hypo-osmolar stress can affect cell processes including signal transduction, ion homeostasis, volume regulatory processes, cytoskeletal organisation, cell cycle and energy metabolism, with a significant proportion of regulatory processes common to both types of stress^28^. Changes in volume and shape have been shown to contribute to GBM active migration through brain tissue^29^. GBM cells benefit from a number of mechanisms allowing them cells to reduce their volume, including volume activated chloride currents^30,31^. In *in vitro* experiments, hyperosmotic stress by addition of dextran molecules to the culture medium was shown to trigger morphological transition into an elongated (or lower circularity ratio) shape^32^. We also tested the effect of hypo-osmotic stress on pro-invasive parameters in light of previous research showing that GBM cells swell in response to hypoxia^33^ and that they withstand cell swelling in response to extreme hypoosmotic stress^34^.

Our experiments show that hyper- and hypo-osmolarity reduced cell viability at early time points but the cells seemed to recover by the 48 h time point (Supplementary Fig. S1). A biphasic effect of osmotic stress on cell cycle has been studied, albeit at different time points: in hypo-osmotic or hyperosmotic cell culture medium, T98G adherent GBM cells showed an increased fraction of cells in S phase, and a decreased fraction of cells in G1 phase compared to cells grown in isotonic medium at 3 days^32^. However, this figure was reversed after 6 days^32^. Our results indicate a minimal impact of hypo-osmolar medium exposure on matrix-degrading enzymes and EMT marker expression (except for an increase in MMP2 in U87, and MMP9 in U251, Fig. 2b). The time course of cell response to swelling may not match the time point at which we analysed the conditioned medium for proteases and the cell lysates for mRNA expression of markers (i.e. 48 h).

Although our results are not uniformly showing an increase of each of the invasive markers tested in all of the cell lines tested, the increase in uPA and/or gelatinase seen in some of the GBM cells exposed to osmotic or hydrostatic pressure is in agreement with previous results showing that GBM cells placed in agarose hydrogels and exposed to 50% compressive strain showed increased mRNA expression of both uPA and uPA receptor by a factor 2–3, as well as cathepsin B and PAI2^35^. In contrast, transcriptional profiling of patient-derived GBM showed that compression (30%) altered the expression of numerous genes, especially those involved in translational control, stress response and solute transport, however neither uPA nor gelatinases were found to be increased at the mRNA level in that study^24^. Our work indicates that GBM reacts to pressure by an increased ability to invade. This could be important in the clinic, with alleviating the pressure, or targeting the mechanism(s) by which pressure promotes GBM invasiveness, as approaches to GBM therapy instead of inhibiting the production or activity of key matrix-degrading enzymes^36^. This is a realistic approach since decreased intracranial pressure can be achieved by antiangiogenic therapies or high dose dexamethasone^8^.

Our experiments show qualitative and quantitative differences in the response to pressure between oncospheres and adherent cell lines. Oncospheres derived directly from primary GBM are known to differ from adherent cell lines and are proposed to better recapitulate the gene expression patterns and *in vivo* biology of human tumours^37^. Furthermore, their response to pressure may be affected by the fact that they grow as floating clumps. When adherent cell lines are exposed to pressure as a monolayer on a culture dish or flask, it is reasonable to speculate that the entire cell surface would be exposed to the treatment. In contrast, the morphology of cells in neurospheres (i.e. an aggregate or cluster of multiple cells) tends towards large clusters, which may restrict exposure to treatment to cells at the surface of the cluster. Overall, both the adherent cell lines and the oncospheres present disadvantages that limit the extrapolation of our results to *in situ* GBM, but their combined use mitigates these limitations. Future experiments will unveil the mechanism(s) transforming the pressure cues into a signalling program that increases invasiveness. Candidate mechanosensory proteins include integrins, growth factor receptors, stretch activated ion channels^38^ aquaporins and other solute transport molecules whose expression is dysregulated in GBM^39^, and caveola-forming proteins^40,41^.

## Methods

### Materials

RPMI-1640 medium, Dulbecco’s Modified Eagle Medium, trypsin-EDTA, penicillin/streptomycin, Coomassie brilliant blue R-250, Pierce BCA Protein Assay Kit and real-time PCR reagents were purchased from Life Technologies (Melbourne, VIC, Australia). The 40% acrylamide/bis solution was from Bio-Rad (Gladesville, NSW, Australia). CultreCoat 24 well plates with BME-coated inserts and CultreCoat 96 well medium BME cell invasion assay kits were from Bio Scientific Pty. Ltd (Sydney, NSW, Australia). NeuroCult NS-A proliferation kit (human), heparin solution, human recombinant bFGF and human recombinant EGF were purchased from Stemcell Technologies Australia (Tullamarine, VIC, Australia). Other reagents were purchased from Sigma-Aldrich (Castle Hill, NSW, Australia) unless otherwise specified.

### Cell culture

Human adherent GBM cell lines U87 and U251 were cultured in RPMI medium supplemented with 5% (v/v) FBS, 100 U/ml penicillin and 100 μg/ml streptomycin. Human prostate cancer DU145 cell line was cultured in RPMI medium with 10% (v/v) FBS, 100 U/ml penicillin and 100 μg/ml streptomycin. Human breast cancer cell lines MDA-MB-231 and MDA-MB-468 were cultured in DMEM medium supplemented with 10% (v/v) FBS, 100 U/ml penicillin and 100 μg/ml streptomycin. Human neurosphere cell lines 081024, JHH136 and 2010.016 A were cultured in NeuroCult NS-A Proliferation medium with 0.2% (v/v) heparin, 20 ng/ml EGF and 10 ng/ml FGF. All cell lines were incubated at 37 °C in a humidified atmosphere with 5% CO_2_.

### Osmolality measurement

The different osmolality media were prepared by adding different volumes of sterile-filtered 5 mol/L NaCl or sterile water. The osmolality of resulting media was measured using an Osmomat 3000 basic freezing point osmometer (Gallay) calibrated using 300 mOsmol/kg and 500 mOsmol/kg standards.

### Osmotic stress

1.0 × 10^6^ cells were seeded in 12 well plates and incubated at 37 °C with 5% CO_2_ for 24 hours. After 24 hours, cells were rinsed twice with serum-free medium and 1 ml of serum-free medium of different osmolality was added to each well and incubated for 48 h. The medium was collected and centrifuged at 1,000 × rpm for 5 min, then stored at −80 °C until analysis.

### Hydrostatic pressure treatment

5.0 × 10^6^ cells were seeded in T25 flasks and incubated at 37 °C with 5% CO_2_ for 24 hours. Cells were rinsed with serum-free medium twice and incubated in 3 ml of serum-free medium containing 4-(2-hydroxyethyl)-1-piperazineethanesulfonic acid (HEPES) at a final concentration of 25 μM. The flask screwcap was fitted with a three-way stopcock and a sphygmomanometer was used to increase the pressure inside the flask to 30 mmHg. The pressure was verified using the sphygmomanometer at the end of the 48 h incubation. Flasks with a ventilated cap were used as unpressured control. The medium was collected and centrifuged at 1,000 × rpm for 5 min, then stored at −80 °C until analysis.

### MTT assay

The cell viability was tested using the 3-[4,5-dimethylthiazole-2-yl]-2,5-diphenyltetrazolium bromide (MTT) assay as previous described^42^. Background absorbance was subtracted and results expressed as the % viability of control cells.

### In gel zymography

The production of uPA, MMP-2 and MMP-9 in response to pressure treatment were measured by casein-plasminogen zymography and gelatin zymography as previous described^40^. Equal protein amounts of conditioned media were loaded in each well. The gels were scanned and uPA, MMP-2 and MMP-9 were quantified by densitometry using Image J (v1.48) software.

### Quantitative RT-PCR

The mRNA expression of specific genes and epithelial to mesenchymal transition (EMT) markers was measured by real-time reverse transcriptase polymerase chain reaction (real time RT-PCR) as previously described^40^. The primers of target genes were TaqMan gene expression assay for human PLAU (Hs01547054_m1), MMP-2 (Hs01548727_m1), MMP-9 (Hs00957562_m1), Snail-1 (Hs00195591_m1), Snail-2 (Hs00161904_m1), N-cadherin (Hs00983056_m1), Twist (Hs01675818_s1), Vimentin (Hs00185584_m1), ZEB1 (Hs01566408_m1) and ZEB2 (Hs00207691_m1). Relative quantification was done by reference to 18S ribosomal RNA (18S rRNA) and analysed using the comparative critical threshold (Ct) method^43^.

### Cell invasion assay

Cell invasion was determined using CultreCoat 24 well plates with BME-coated Inserts as previously described^44^. The crystal violet-stained cells were imaged using a Leica DFC 295 microscope (10×/0.22) with LAS V4.5 software. The invaded cells were counted by Image J (v1.48) software. Alternatively, cell invasion was determined using CultreCoat 96 well medium BME cell Invasion assay with 2% (V/V) serum in the bottom well as per manufacturer’s instructions. The same osmolality was applied in both the upper and lower chambers.

### Statistical analysis

Statistical analysis was carried out using GraphPad Prism software (v. 8.01). P-value of <0.05 was considered significant. All the data are shown as mean ± SEM and show either replicates or independent experiments as detailed in the figure legends.

## Supplementary information

supplementary information.
