# Supplementary material for: Matrix protease production, epithelial-to-mesenchymal transition marker expression and invasion of glioblastoma cells in response to osmotic or hydrostatic pressure
Source: Sci Rep. 2020 Feb 14;10:2634. doi: 10.1038/s41598-020-59462-w (PMC7021835; doi:10.1038/s41598-020-59462-w)
Supplement: Supplementary file 1 — supplementary information. [file 41598_2020_59462_MOESM1_ESM.pdf]

**Supplementary data for :**

# Matrix protease production, epithelial-to-mesenchymal transition marker expression and invasion of glioblastoma cells in response to osmotic or hydrostatic pressure.

Wenjun Pu, Jiawen Qiu, Gregory J. Riggins, Marie-Odile Parat

Supplementary figure 1

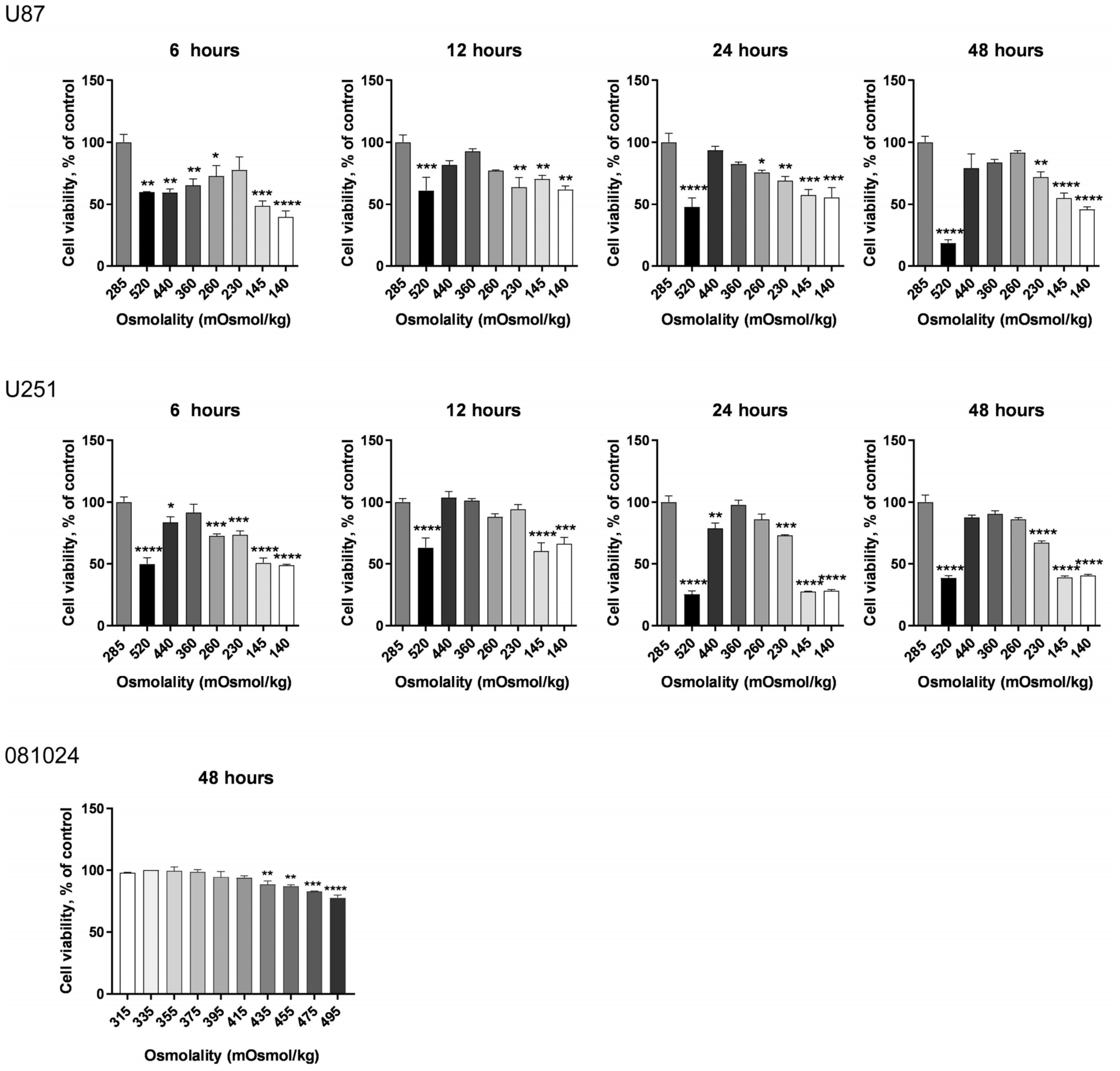


**Supplementary Figure 1** The cell viability of GBM adherent and neurosphere cell lines was tested after incubation in media of the indicated osmolality using the MTT assay. Results are expressed as percent of the viability of control cells incubated in normo-osmotic medium (285 mOsmol/kg for U87 and U251 cells, and 315 mOsmol/kg for oncospheres). Viability of the U87 and U251 cells was measured at 6, 12, 24 and 48 hours. Viability of the 081024 cell was measured at 48 hours. Data are shown as the mean ± SEM of n = 3 independent experiments, * $p < 0.05$, ** $p < 0.01$, *** $p < 0.001$, **** $p < 0.0001$, one way ANOVA analysis with Dunnett's multiple comparisons test.

Supplementary Figure 2

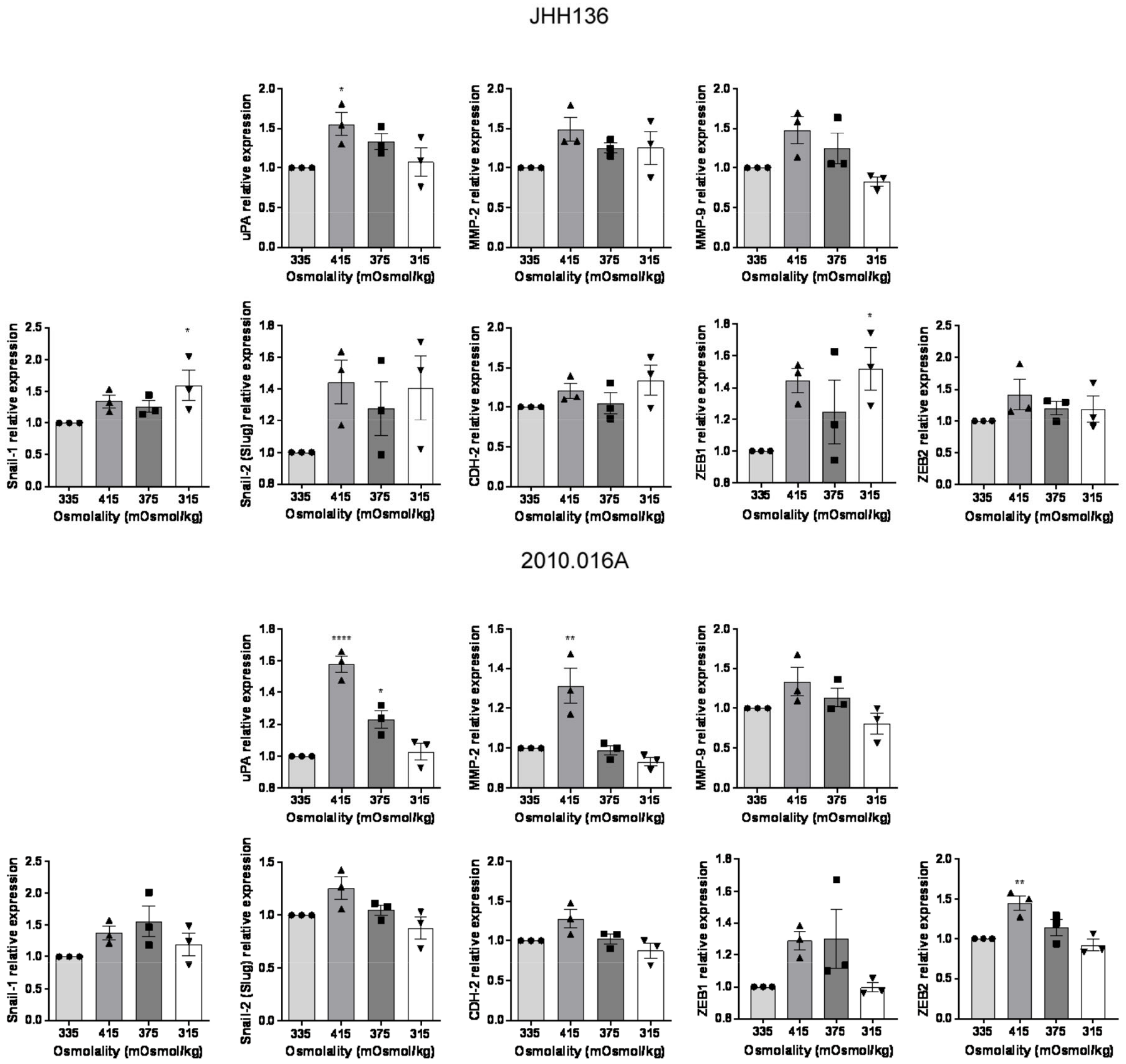


**Supplementary figure 2:** mRNA expression of proteases uPA, MMP-2 and MMP-9 and EMT markers Snail-1, Slug, CDH-2, ZEB1 znd ZEB2 in oncosphere cell lines JHH 136 and 2010.016A in control (335 mOsmol/kg) hyper (415 or 375 mOsmol/kg) or hypoosmotic stress (315 mOsmol/kg). All results are expressed as mean ± SEM of n = 3 independent experiments, * p <0.05, ** p <0.01, *** p <0.001, **** p <0.0001, one way ANOVA analysis with Dunnett's multiple comparisons test.

Supplementary figure 3

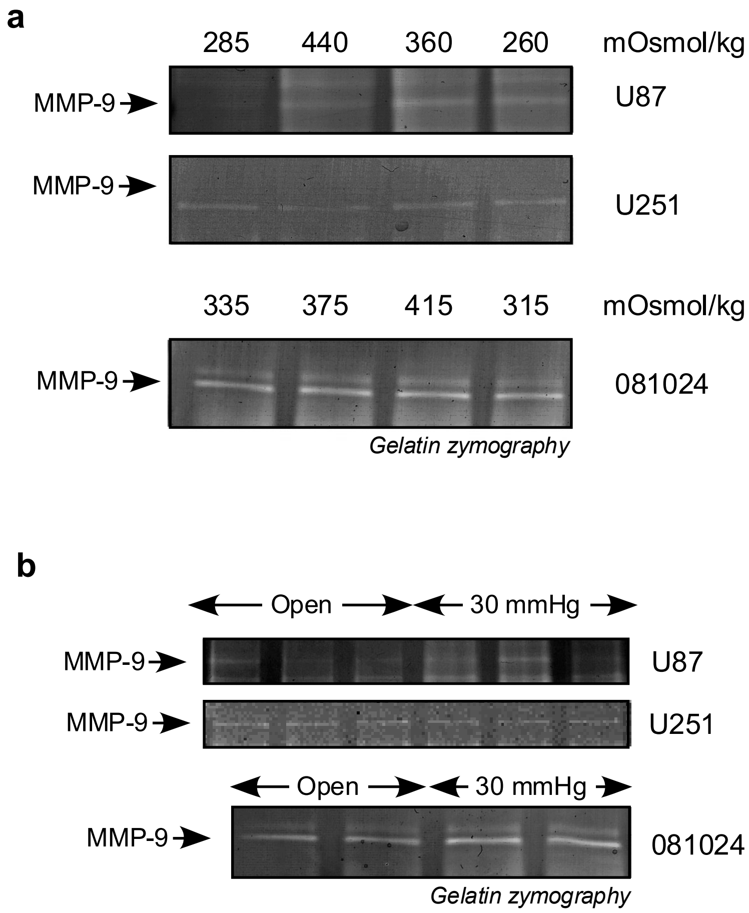


**Supplementary Figure 3** Effect of osmotic or hydrostatic pressure on MMP-9 production by GBM adherent and neurosphere cells. A) Conditioned media of cells exposed to osmotic stress were analysed by gelatin zymography. Control medium is 285 mOsmol/kg for U87 and U251 cell lines, and 335 mOsmol/kg for oncospheres. B) Conditioned media of cells exposed to hydrostatic pressure for 48 h were analysed by gelatin zymography.

Supplementary figure 4

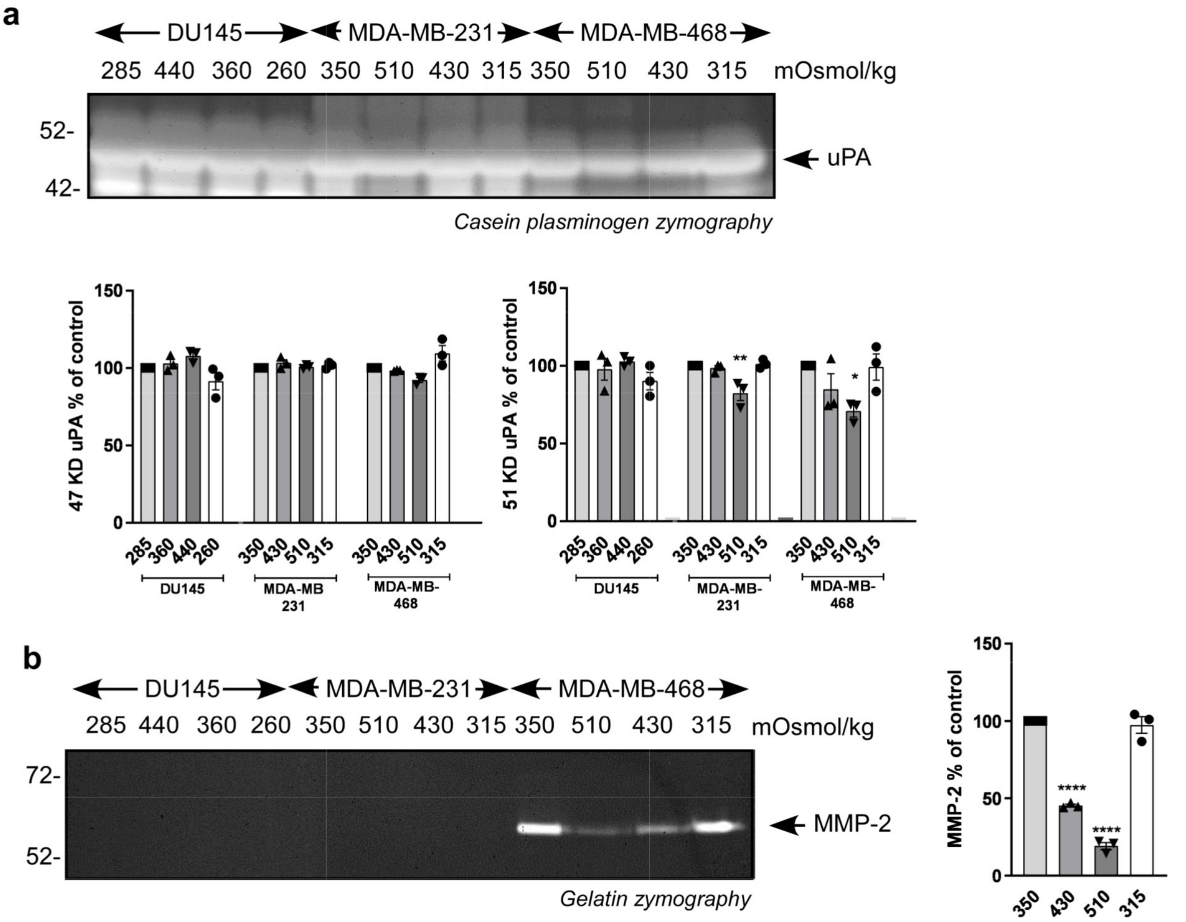


**Supplementary Figure 4** Effect of osmotic pressure on non-GBM cancer cell lines. Control medium is 285 mOsmol/kg for DU145, and 350 mOsmol/kg for MDA-MB-231 and 468 cells. A) Conditioned media of cells exposed to hydrostatic pressure for 48 h were analysed by casein plasminogen zymography and the 47 KD and 51 KD bands corresponding to uPA were quantitated. B) Conditioned media of cells exposed to hydrostatic pressure for 48 h were analysed by gelatin zymography. Densitometric quantitation of MMP-2 produced in the conditioned medium is shown. All results are expressed as mean ± SEM of n = 3 independent experiments, * $p < 0.05$, ** $p < 0.01$, ****$p < 0.0001$, one way ANOVA analysis with Dunnett's multiple comparisons test.

Supplementary figure 5

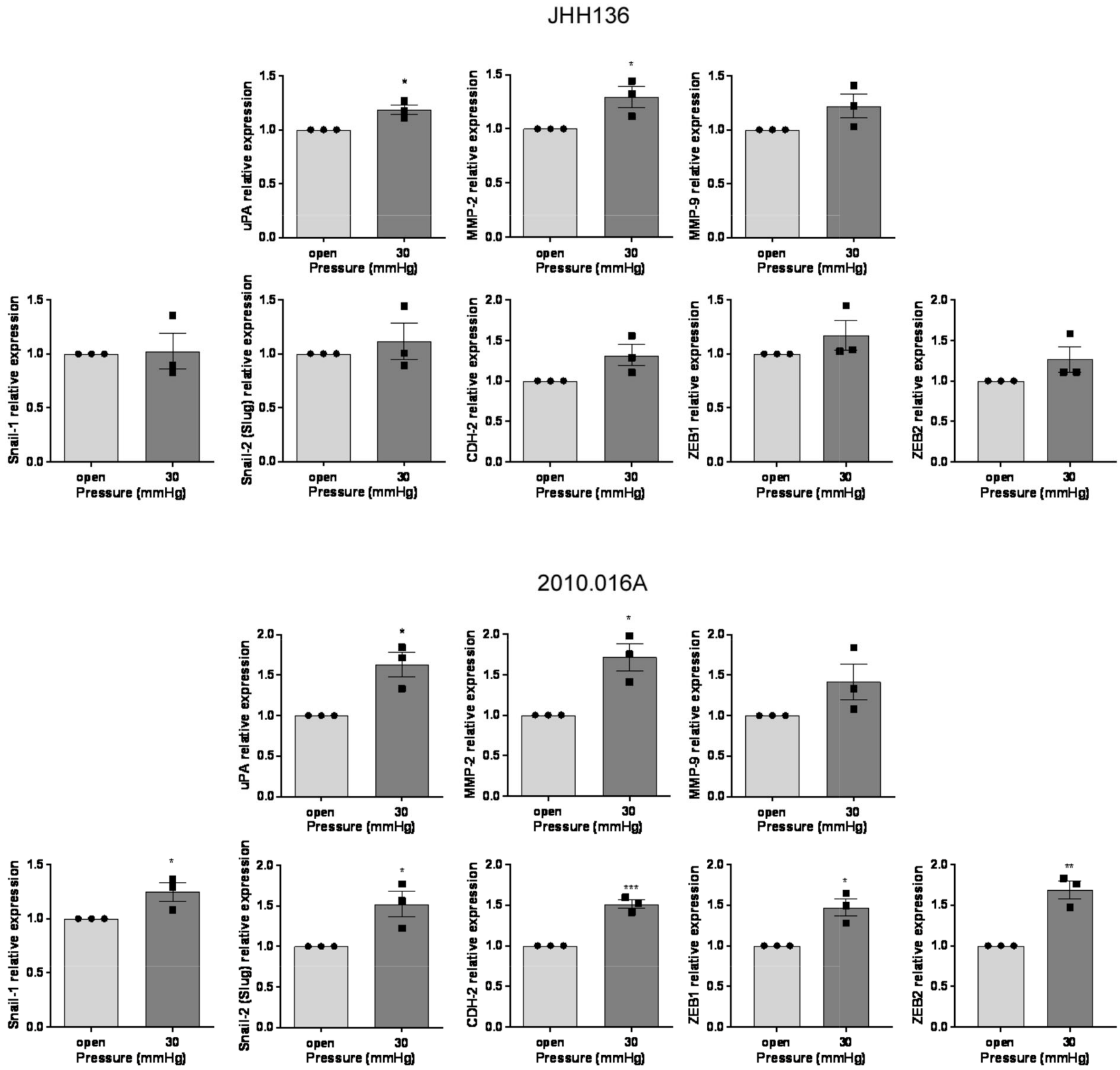


**Supplementary figure 5:** Effect of hydrostatic pressure on mRNA expression of proteases uPA, MMP-2 and MMP-9; and EMT markers Snail-1, Slug, CDH-2, ZEB1 znd ZEB2 in oncosphere cell lines JHH 136 and 2010.016A. All results are expressed as mean ± SEM of n = 3 independent experiments, * p <0.05, ** p <0.01, *** p <0.001, **** p <0.0001, one way ANOVA analysis with Dunnett's multiple comparisons test.

Supplementary figure 6

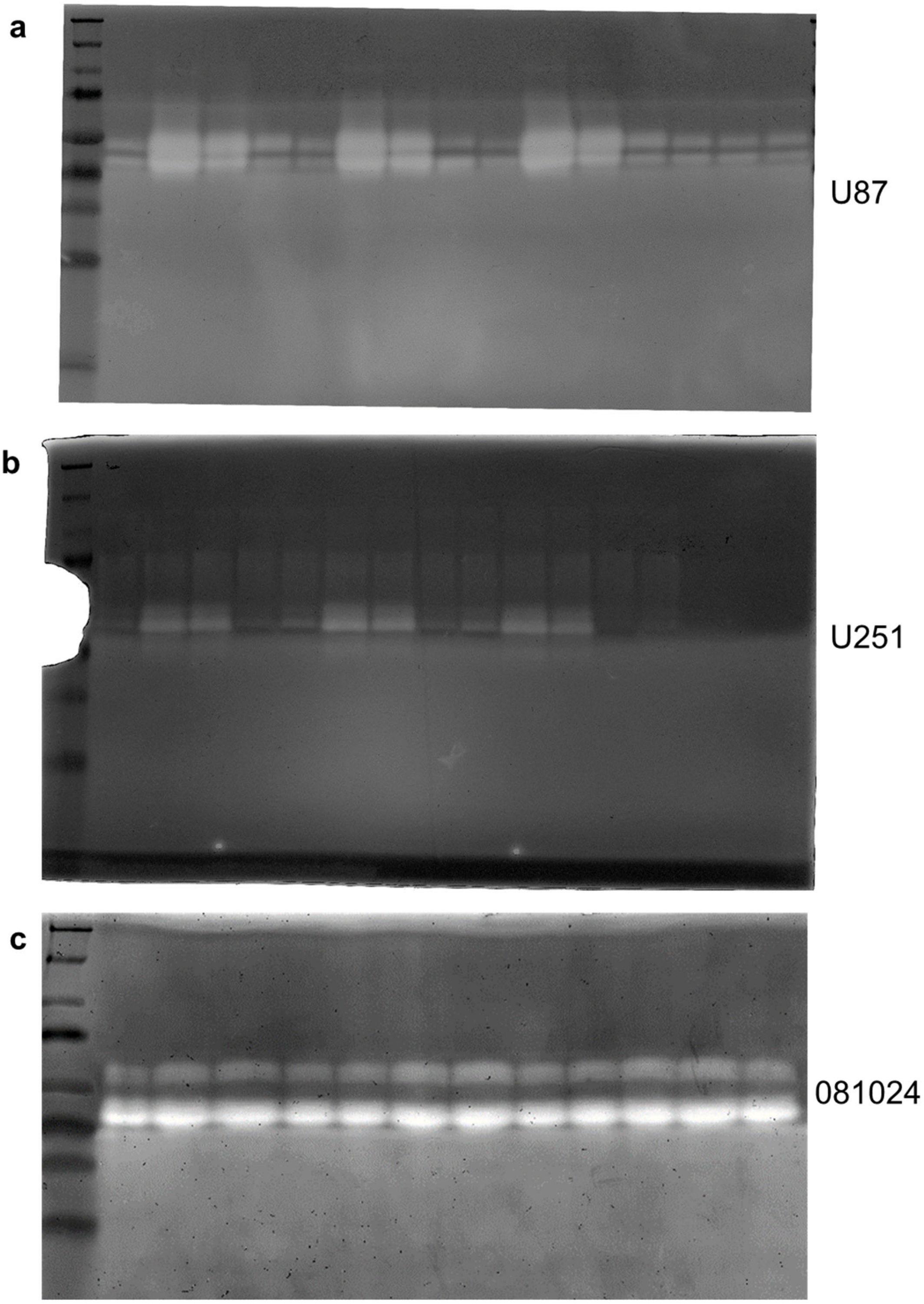


**Supplementary Figure 6** Original gel pictures for Fig. 1. a, b, and c with samples from U87, U251 and 081024 cells.

Supplementary figure 7

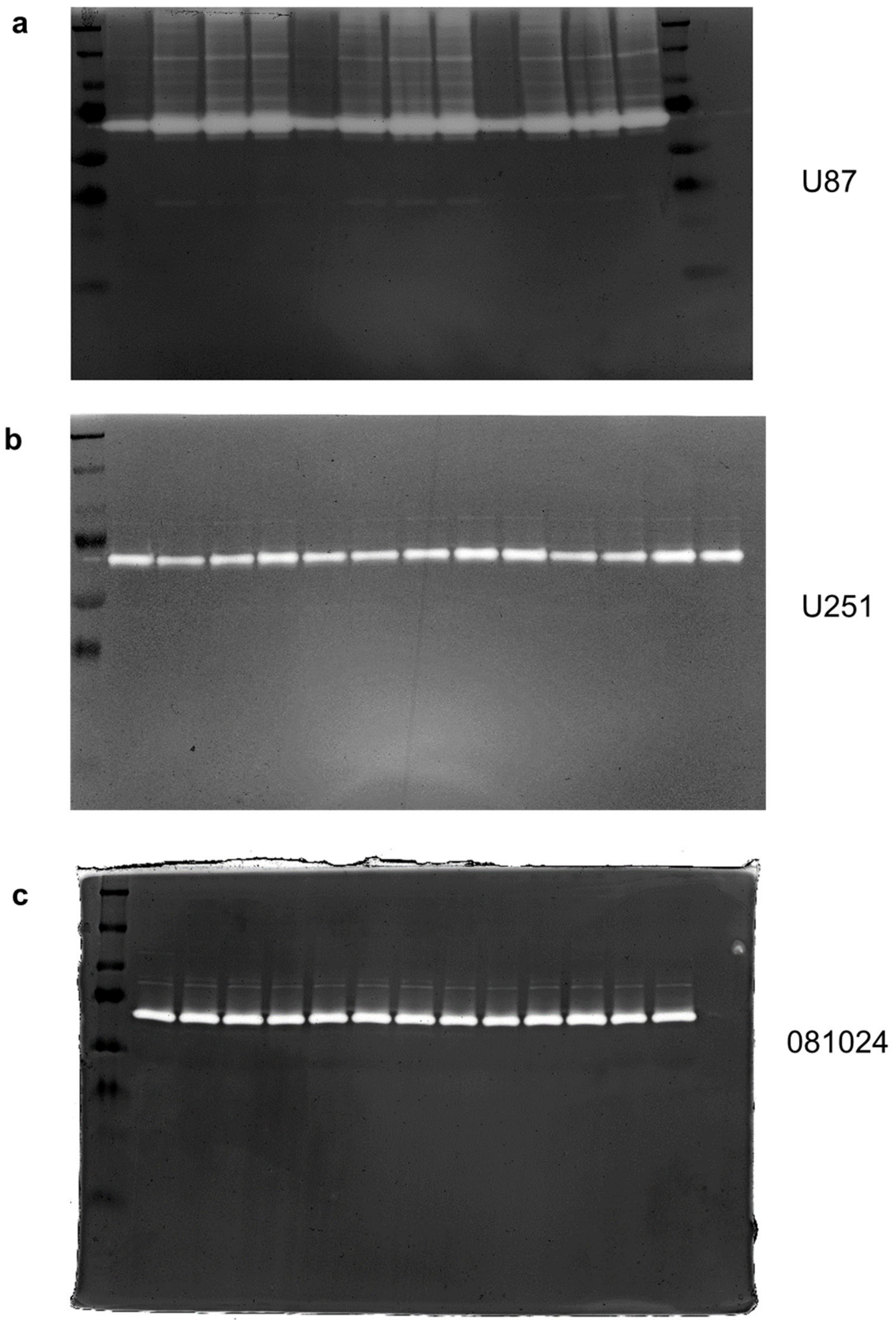


**Supplementary Figure 7** Original gel pictures for Fig. 2. a, b, and c with samples from U87, U251 and 081024 cells.

Supplementary figure 8

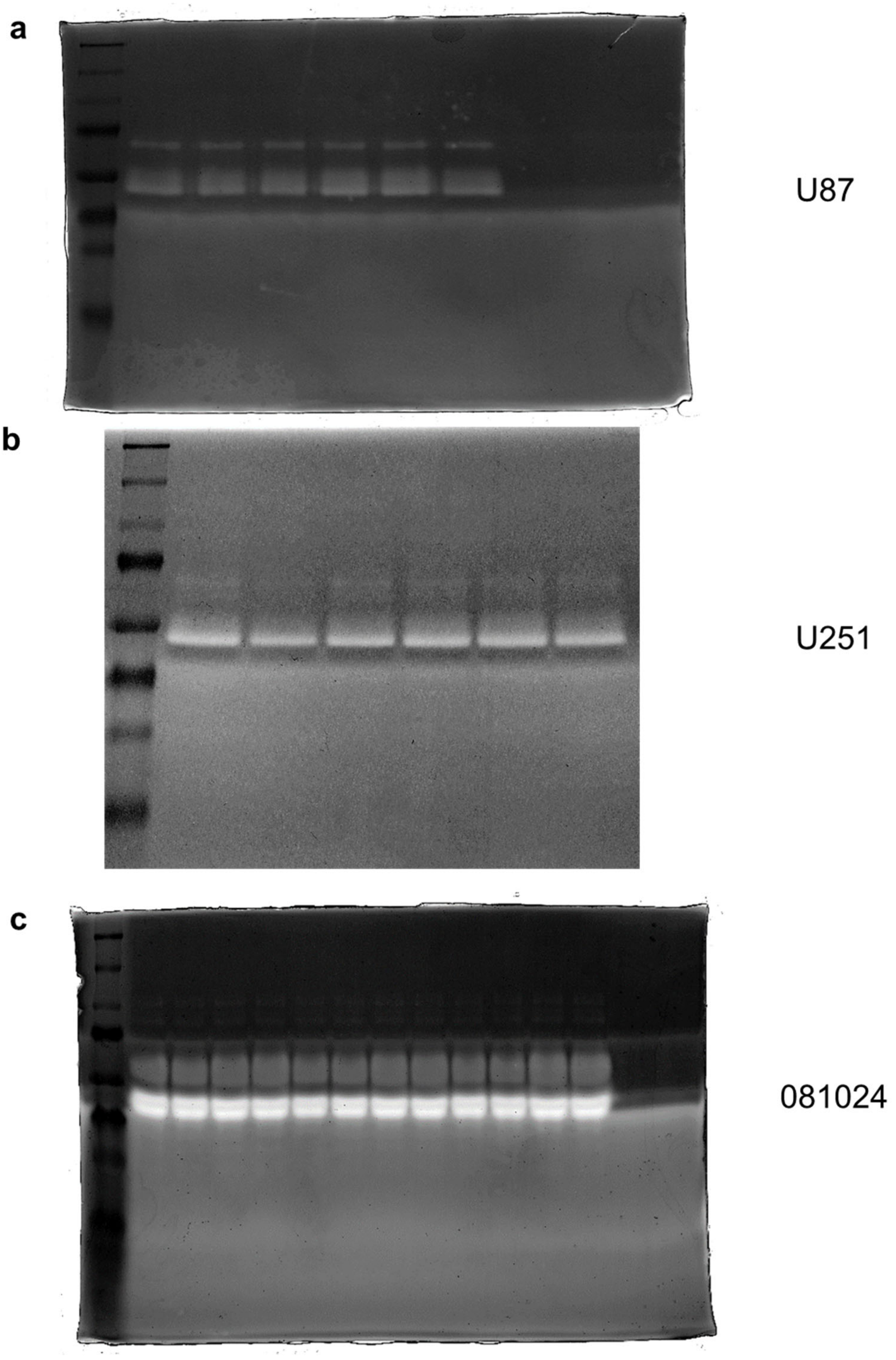


**Supplementary Figure 8** Original gel pictures for Fig. 4. a, b, and c with samples from U87, U251 and 081024 cells.

Supplementary figure 9

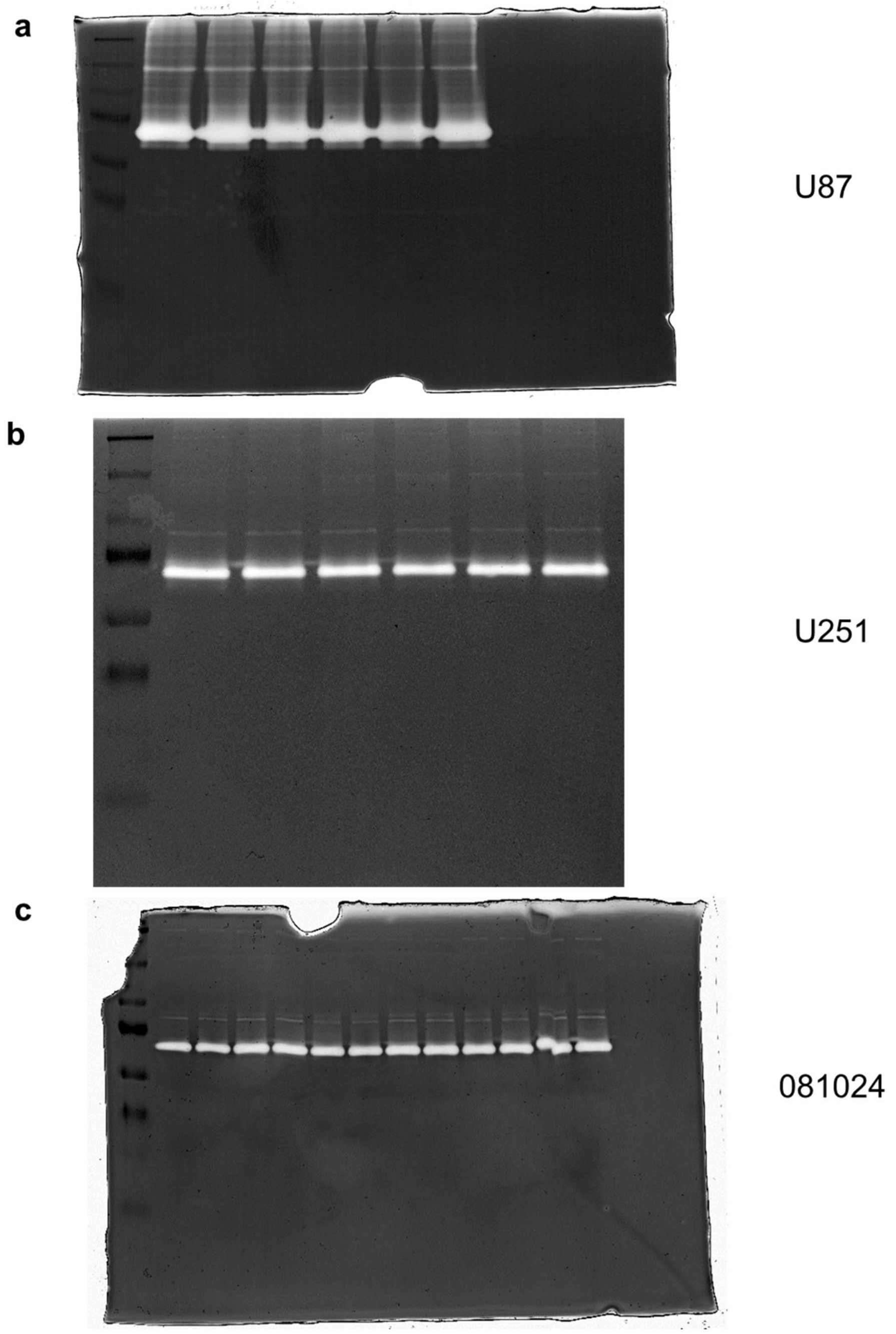


**Supplementary Figure 9** Original gel pictures for Fig. 5. a, b, and c with samples from U87, U251 and 081024 cells.

Supplementary figure 10

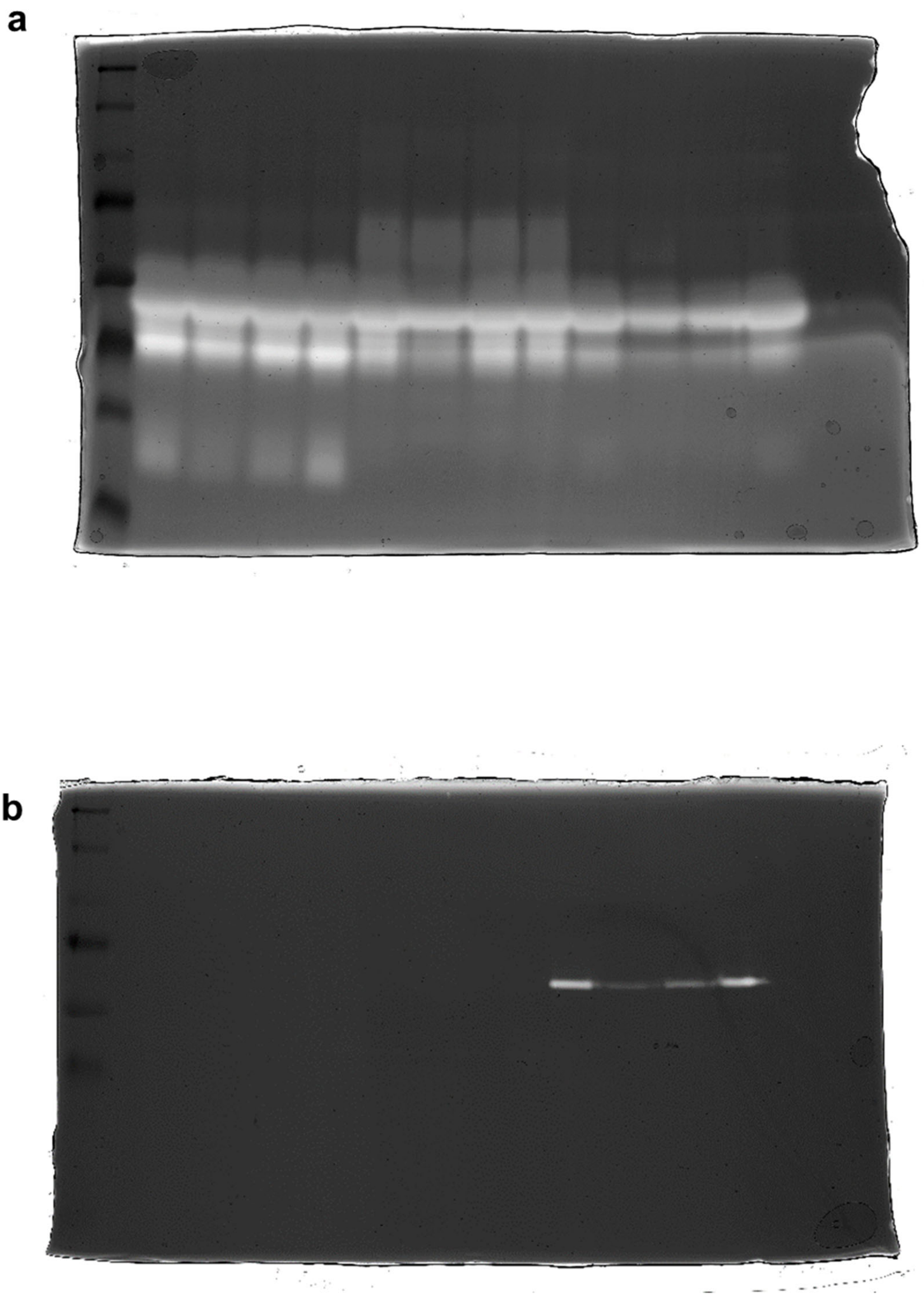


**Supplementary Figure 10** Original gel pictures for supplementary Fig. S4. a and b with casein plasminogen and gelatin zymography, respectively.
